# Efficacy of treatment for subacromial pain in older adults: a systematic review and meta-analysis with GRADE recommendations

**DOI:** 10.1007/s41999-026-01511-0

**Published:** 2026-05-28

**Authors:** Gabriel de Castro Mariz, Diego Mendes Xavier, Micaelen Mara Ferreira Barroso, Alessandra de Carvalho Bastone, Arthur Nascimento Arrieiro, Vinicius Cunha de Oliveira, Murilo Xavier de Oliveira

**Affiliations:** Department of Physiotherapy, Universidade Federal Dos Vales Do Jequitinhonha E Mucuri (UFVJM), Campus JK, Rodovia MG 367, Km 583, nº 5000-Alto da Jacubá, Diamantina, MG 39100-000 Brazil

**Keywords:** Subacromial pain, Older adults, Treatment efficacy

## Abstract

**Abstract:**

Subacromial pain is a prevalent musculoskeletal condition that impairs shoulder function, particularly in older adults. It is often associated with tendinopathy, bursitis, and biomechanical dysfunction, leading to limitations in daily activities and decreased quality of life. Despite its clinical relevance, studies targeting individuals aged 60 years and older are scarce. The aim of this review was to systematically synthesize the evidence from randomized controlled trials (RCTs) evaluating the effectiveness of conservative treatments for subacromial pain in adults aged 60 years and older.

**Methods:**

This review followed the Cochrane and PRISMA guidelines and was registered in PROSPERO (CRD42024591488). Six databases were searched without restrictions. Eligible RCTs compared therapeutic interventions to minimal intervention controls and reported outcomes on pain and/or disability. Risk of bias was assessed using the PEDro scale, and the certainty of evidence was rated using the GRADE approach. Meta-analyses were performed using random-effects models.

**Results:**

Out of 6,198 records, four RCTs met the inclusion criteria. Interventions included resistance training, cycle ergometer exercise, dry needling, and osteopathic manipulative treatment. The meta-analysis of three trials showed a statistically significant pooled effect for pain reduction (MD = −1.60; 95% CI: −3.16 to −0.04); however, two studies (resistance training and cycle ergometer) demonstrated significant individual benefits. The overall certainty of evidence ranged from low to high. No study assessed disability using validated upper limb-specific instruments.

**Conclusions:**

Although some interventions demonstrated positive effects on pain, the overall evidence for conservative treatments in older adults with subacromial pain is limited. Further high-quality trials are needed, focusing on functional outcomes and long-term benefits.

**Supplementary Information:**

The online version contains supplementary material available at https://doi.org/10.1007/s41999-026-01511-0.

## Key summary points

**Aim:**

This systematic review and meta-analysis primarily aimed to evaluate the effectiveness of conservative treatments for subacromial pain in adults aged 60 years and older.

**Findings:**

Across four randomized controlled trials, conservative interventions showed a statistically significant pooled effect for pain reduction, although the proximity of the confidence interval to the null suggests the results should be interpreted with caution. Notably, no study assessed disability using validated upper limb-specific instruments.

**Message:**

The limited overall evidence for conservative treatments in this older population highlights the critical need for further high-quality trials that focus on functional outcomes and long-term benefits.

**Supplementary Information:**

The online version contains supplementary material available at https://doi.org/10.1007/s41999-026-01511-0.

## Introduction

Subacromial pain is a highly prevalent source of shoulder discomfort, often stemming from rotator cuff tendinopathy, subacromial bursitis, or partial tendon tears. Clinically, it is strongly associated with biomechanical alterations, muscle weakness, and physical inactivity [1]. While this condition affects individuals across the lifespan, it imposes a particularly severe burden on older adults. In this population, the risk is exacerbated by age-related musculoskeletal decline, cumulative tissue degeneration, and prolonged sedentary behaviors [2].

The clinical and personal impact of subacromial pain in the older people is profound. It significantly restricts basic activities of daily living (ADLs), such as grooming and bathing, and compromises independence in more complex instrumental tasks, including housekeeping and grocery shopping, thereby drastically reducing overall quality of life [2]. Notably, shoulder pain ranks as the third most common chronic musculoskeletal complaint, surpassed only by low back and knee pain [3]. Yet, despite its high prevalence and debilitating nature, research focused specifically on the geriatric population remains surprisingly scarce.

Current literature broadly supports conservative management—such as physiotherapy and corticosteroid injections—for adults with subacromial pain. However, most clinical trials encompass wide age cohorts and rarely perform age-stratified analyses to isolate outcomes for older adults. Consequently, while interventions like resistance exercises [4], flexibility training [5], dry needling [6], and joint mobilization [7] show general promise, their specific efficacy, safety, and optimal application in patients over 60 years old remain critically under-investigated.

Addressing this distinct gap in the literature is essential for developing targeted, evidence-based clinical guidelines for a rapidly aging population. Therefore, this study aimed to systematically review and synthesize the current evidence from randomized controlled trials evaluating the effectiveness of conservative treatments for subacromial pain specifically in individuals aged 60 years and older. Furthermore, to ensure the reliability of our findings for clinical decision-making, we applied the GRADE approach to rigorously evaluate the certainty of the synthesized evidence.

## Methods

This study was conducted following Cochrane recommendations for systematic reviews [8] and reported according to the PRISMA checklist [9] (Appendix 1). The protocol was prospectively registered in PROSPERO CRD42024591488.

### Search strategies and sources

Searches were conducted in the MEDLINE, Cochrane, EMBASE, AMED, PsycInfo, and PEDro databases, with no restrictions regarding language or year of publication. The search terms were “randomized controlled trials,” “shoulder pain,” and “older adults”. The search was performed on July 12, 2023 (updated July 22, 2025). The detailed search strategy is provided in Appendix 2. Reference lists from relevant systematic reviews in the field were also manually searched to identify potentially relevant studies that may have been missed in the initial search. As of the publication date, no new studies have been identified. The PICOT research question is detailed in Appendix 3.

### Inclusion criteria

Eligible studies included parallel, crossover, or cluster randomized controlled trials (RCTs) that examined the efficacy or additional effects of treatments in individuals 60 years of age or older with subacromial pain. Trials were considered eligible if both male and female participants from any healthcare setting were enrolled. The comparator groups in these trials were defined as minimal intervention controls (e.g., no intervention, waiting list, placebo, or sham) to assess the efficacy of the treatment. The primary outcomes of interest were disability and pain intensity, with valid questionnaires or scales used to measure these outcomes [10].

Conversely, studies were excluded if they evaluated surgical procedures or pharmacological interventions, as our focus is strictly on conservative treatments. Furthermore, we excluded studies involving participants with other specific shoulder pathologies (e.g., fractures, adhesive capsulitis, or severe glenohumeral osteoarthritis), non-randomized study designs, and studies where data for the subpopulation aged 60 years or older could not be extracted separately.

### Selection of trials

After conducting the searches, the references were exported to the Rayyan web app (https://www.rayyan.ai/) and duplicates were removed. Two independent reviewers (GDCM and MMFB) screened the titles and abstracts, followed by an analysis of the full texts. Studies meeting the eligibility criteria were included in the review. Divergences of opinion between the reviewers were resolved by a third reviewer (VCDO).

### Data extraction

Two independent reviewers (GDCM and MMFB) extracted data on the study characteristics and outcomes from the trials included in the review. Divergences of opinion between the reviewers were resolved by a third reviewer (VCDO). The extracted data were study design, participant characteristics (age, source), type of chronic pain, intervention and comparator details, outcomes, and time points. For the outcomes of interest, we extracted post-intervention means, standard deviations (SDs), and sample sizes for all groups at the following time points: immediate, short-term, medium-term, and long-term effects. The immediate effect was considered the outcome immediately after treatment. The short-term effect was defined as follow-up within three months after baseline. The medium-term effect was defined as follow-up between three and 12 months and the long-term effect was defined as follow-up at least 12 months after baseline. If multiple time points were available within the same follow-up period, the time point closest to the end of the intervention was chosen for analysis [11].

### Handling missing data

In instances of missing data, we made three separate attempts spaced one week apart to contact the original investigators and request the necessary information. When the mean and standard deviation or 95% confidence intervals could not be obtained, we applied established imputation methods recommended in the GRADE and Cochrane guidelines [12]. Specifically, we assumed that the median approximated the mean, a method considered acceptable in approximately symmetric distributions [13, 14]. For the standard deviation, we used the formula that estimates the standard deviation as the interquartile range (IQR) divided by 1.35, based on the properties of a normal distribution. This approximation is widely used in meta-analyses when only non-parametric data are available and is supported by simulation studies demonstrating acceptable bias under conditions of moderate skewness [15]. These procedures allowed us to include data that would otherwise be excluded, reducing the risk of bias due to incomplete outcome reporting.

### Appraisal of methodological quality of trials included

Two independent reviewers (GDCM and MMFB) assessed the methodological quality of the included randomized controlled trials using the Physiotherapy Evidence Database (PEDro) scale, a validated and widely used tool for evaluating the internal validity and interpretability of physiotherapy trials. The PEDro scale consists of 11 items, of which 10 are scored, resulting in a total score ranging from 0 to 10. Studies were classified as having high methodological quality (scores ≥ 6), moderate quality (scores 4–5), and low quality (scores ≤ 3), based on criteria proposed by Moseley et al. [16]. When available, PEDro scores were obtained directly from the PEDro database. Disagreements between reviewers were resolved by consulting a third reviewer [16].

### Data analysis

Meta-analyses were performed using random-effects models. Mean differences (MDs) and 95% confidence intervals (CIs) were presented in forest plots. For cases in which standardization of the results was not possible due to inconsistent outcome measures, standardized mean differences (SMDs) were employed [17]. All analyses were conducted using the Review Manager 5.3 software (The Nordic Cochrane Centre, Copenhagen, Denmark). Effect sizes of interventions for each outcome were interpreted based on minimal clinically important differences [18], taking into account the confidence intervals of the estimates [19].

Effect sizes of interventions for each outcome were interpreted based on minimal clinically important differences [18], taking into account the confidence intervals of the estimates [19].

### Certainty of evidence

Two independent reviewers (GDCM and MMFB) assessed the certainty of the evidence using the GRADE approach [20]. GRADE rates the certainty of evidence as high, moderate, low, or very low, reflecting the confidence that the estimated effect is close to the true effect. Evidence was initially considered high and downgraded by one level for each of the following domains when concerns were identified: (1) Publication bias, when at least ten trials showed asymmetry suggestive of bias in the analysis [20]; (2) Imprecision, when the total sample size across studies was below 400 participants [21]; (3) Risk of bias, when more than 25% of the participants were enrolled in studies classified as having a high overall risk of bias, as determined by PEDro scores below 6, indicating major methodological limitations such as lack of allocation concealment or blinding [22]; (4) Inconsistency, when heterogeneity was substantial (I^2^ > 50%), pooling was not feasible, or there were clear visual discrepancies among trials in the forest plots [11]. Disagreements between reviewers were resolved through consultation with a third reviewer (VCDO).

## Results

**Fig. 1:**
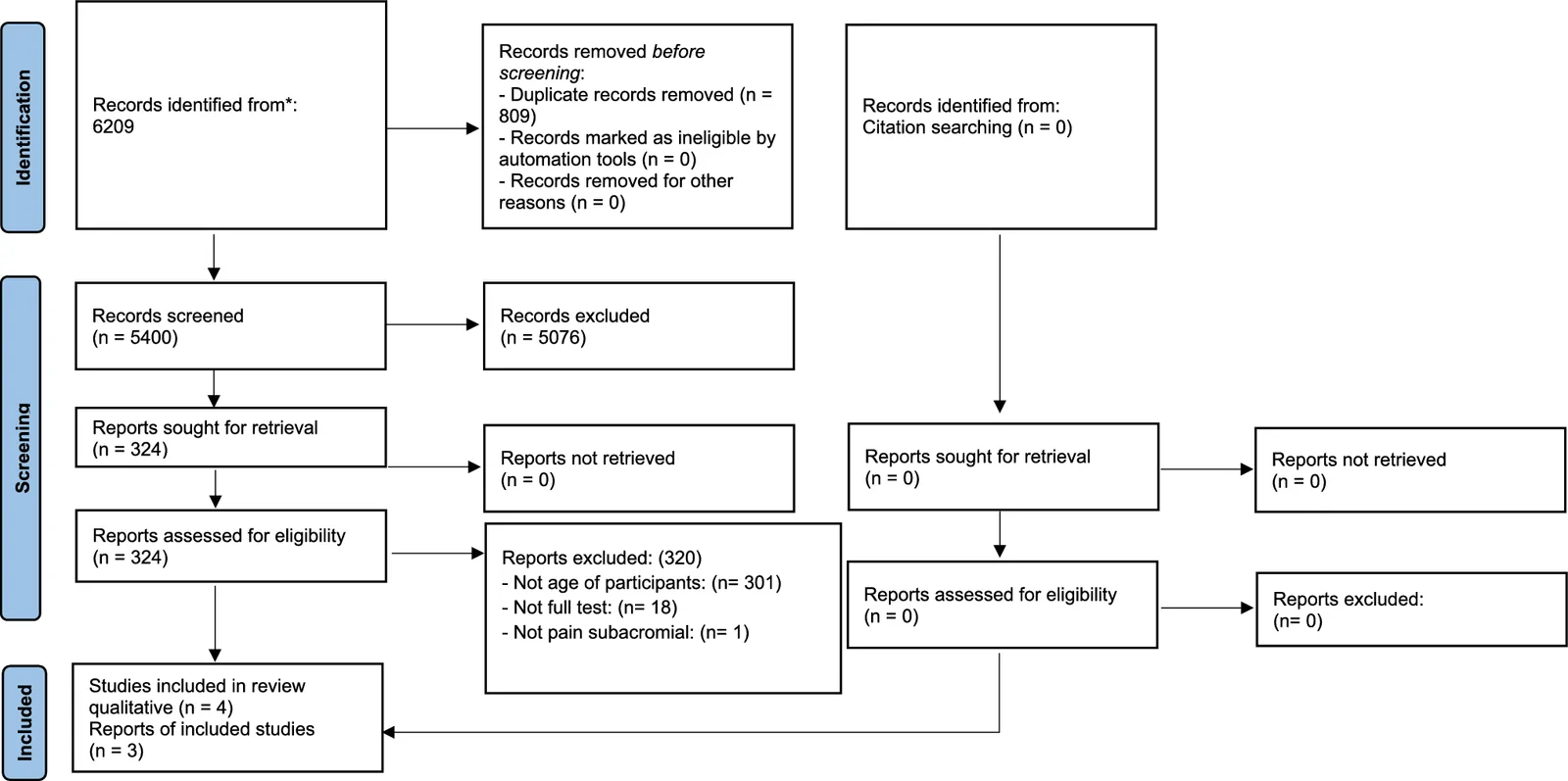
Study flowchart

The electronic search strategy identified a total of 6,198 titles and abstracts. After removing 809 duplicates, 5,389 references were screened, leading to the selection of 324 potentially eligible studies, 320 of which were excluded. Thus, only four studies met the inclusion criteria: [4, 6, 23, 24]. Figure 1 displays the flowchart of the article selection process.

Among the studies included, one investigated the effect of supervised cycle ergometer training on reducing pain during activities, comparing it to a control group that performed home exercises only [23]. Another examined the efficacy of dry needling applied to both a latent and an active motor point in the infraspinatus muscle, using dry needling at the active point only as the comparator [6]. A third study evaluated a resistance exercise program with elastic bands aimed at reducing pain and stress, improving shoulder range of motion (ROM), and enhancing body composition in older adults, with a control group instructed to maintain their usual daily activities [4]. Lastly, Knebl et al. [24] assessed the effectiveness of osteopathic manipulative treatment compared to a sham intervention that mimicked the procedure without the application of therapeutic force. A detailed characterization of the included studies is provided in Table 1.

**Table 1:** Characteristics of the included trials (n = 4)

Study	Health condition	Participants	Intervention	Outcome measures
Gialanella et al. (2018)	Symptomatic full-thickness rotator cuff tear	40 Older outpatients (> 70 years) with rotator cuff tear were randomized to two groups: cycle ergometer (CYC) vs. control patients	All patients underwent a common outpatient rehabilitation exercise program consisting of ten 30-min sessions (5 sessions/week). At discharge, CYC patients received 15-min training with an arm cycle ergometer and were asked to use the device at home 20 min/twice a day. Control patients were asked to continue the standard exercises. During the 6-month study period, CYC patients (but not control patients) received educational reinforcement monthly from the nurse of the telemedicine service	Outcomes assessed between admission (T0) and 6-month follow-up (T6): pre to post pain during activities, active and passive ROMsum, ROM- pain sum, revised Constant Total Score, and Health Assessment Questionnaire (HAQ)
		Average age: CYC group: 79.6 (± 4.1) years		
		Control group: 79.9 (± 4.3) years		
		Sex: CYC group: 100% women		
		Control group: 89.4% women/10.6% men		
Calvo-lobo et al. (2018)	Nonspecific shoulder pain with myofascial trigger points (MTrPs)	Sixty-six patients aged 65 years and older with trigger points in the ipsilateral infraspinatus of the painful shoulder were randomly assigned to 1 of 2 treatment groups	A session of dry needling on the infraspinatus was performed in (1) the most hyperalgesic active and latent MTrP or (2) only the most hyperalgesic active MTrP	The Numeric Rating Scale, pressure pain threshold (primary outcome) on the anterior deltoid and extensor carpi radialis brevis latent MTrPs, and grip strength were assessed before, after, and 1 week after the intervention
		Average age: group 1 (active and latent): 75.35 (± 6.97) years		
	Infraspinatus muscle	Group 2 (active only): 76.74 (± 8.20) years		
		Sex (total): 43 women (65.2%) and 23 men (34.8%)		
Lee et al. (2022)	Shoulder	Participants were 80 adults aged 60 or older residing in the community. Average age and sex: Age: ≥ 60 years (exact data on average ages and sex distribution are not detailed in the publicly available summary)	Participants were randomly assigned using random number generation to an intervention group (n = 40) or control group (n = 40). The intervention group participated in a resistance exercise program using an elastic band three days a week for 12 weeks. The control group followed a daily routine for 12 weeks	Measurements were conducted three times: at baseline, at the sixth week of treatment, and post intervention
	joint muscle—related pain			
Knebl et al. (2002)	Common shoulder problems	Twenty-nine older patients with preexisting shoulder problems. Each participants had chronic pain, decreased ROM, and/or decreased functional ability in the shoulder before entering the study. Average age: not reported as a single value, however the vast majority (93.1%) are over 65 years old. Sex (total): 62.1% women and 37.9% men	Participants were randomly assigned to either a treatment (OMT) group or a control group for 14 weeks. Over the course of treatment, both groups had significantly increased ROM and decreased perceived pain	Functional independence, ROM of the shoulder, and pain over the course of treatment, both groups had significantly increased ROM and decreased perceived pain

The table presents a summary of the methodological characteristics, population evaluated, interventions applied, and outcome measures of the selected studies, focused on the treatment of conditions associated with shoulder pain and dysfunction in older adults

*CYC* cycle ergometer, *HAQ* Health Assessment Questionnaire, *MTrPs* myofascial trigger points, *OMT* osteopathic manipulative treatment, *ROM* range of motion

### Efficacy of treatment on subacromial pain outcomes

For the meta-analysis, the statistics were based on a single common outcome of interest: shoulder joint pain. Only three studies provided mean and standard deviation data for this outcome [4, 6, 23]. The characterization of pain is detailed in Appendix 4.

**Fig. 2:**
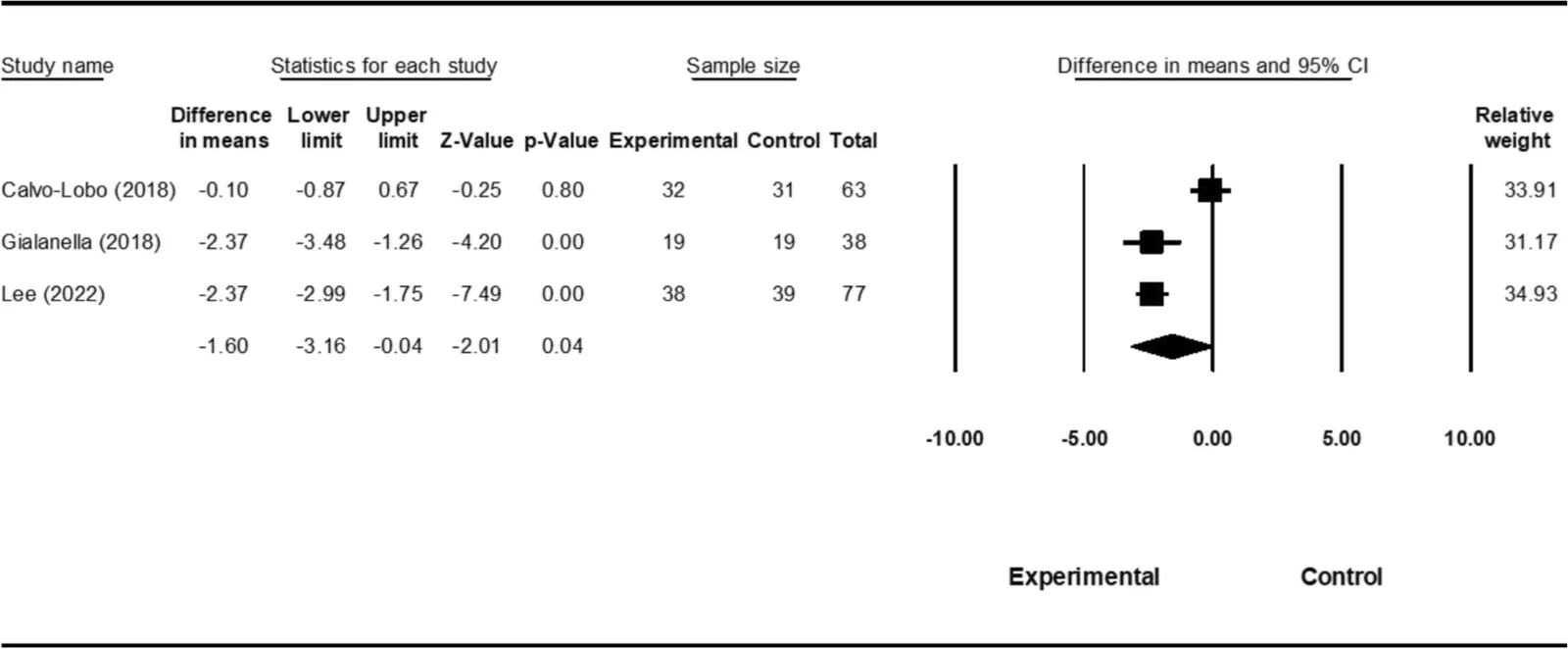
Forest plot of the mean difference in pain intensity between the intervention and control groups

Data from three studies were pooled for the meta-analysis, revealing a statistically significant reduction in pain favoring the intervention group. The forest plot (Fig. 2) illustrates this overall effect, with a mean difference (MD) of −1.60 (95% CI: −3.16 to −0.04; p = 0.04).

However, significant reductions in shoulder pain were found in the studies conducted by Gialanella et al. [23], who evaluated supervised cycle ergometer training (MD: −2.37, CI: −3.48 to −1.26, p = 0.00), and Lee et al. [4], who investigated resistance exercises with elastic bands (MD: −2.37, CI: −2.99 to −1.75, p = 0.00). In contrast, the study by Calvo-Lobo et al. [6], which assessed dry needling for myofascial shoulder pain, did not show significant pain reduction compared to controls (MD: −0.10, CI: −0.87 to 0.67, p = 0.80).

The study by Knebl et al. [24], who assessed the effectiveness of osteopathic manipulative treatment for the shoulder joint, was only included in the qualitative analysis due to the unavailability of sample mean and standard deviation data. The authors reported significant improvements in ROM and a reduction in pain perception.

### Quality appraisal

The results of the quality appraisal of the studies included are provided in Table 2. The studies conducted by Gialanella et al. [23] and Knebl et al. [24] (50%) had a high risk of bias, although with acceptable methodological quality. The studies conducted by Calvo-Lobo et al. [6] and Lee et al. [4] (50%) had a low risk of bias and good methodological quality.

**Table 2:** Quality assessment

Study	Score	Risk of Bias	Quality
Gialanella et al. (2018)	5	Serious	Acceptable quality
Calvo-lobo et al. (2018)	7	Not serious	Good quality
Lee et al. (2022)	8	Not serious	Good quality
Knebl et al. (2002)	4	Serious	Acceptable quality

### GRADE assessment of evidence quality

The assessment using the GRADE system indicated low-quality of evidence for dry needling, moderate certainty for the cycle ergometer, and high certainty for resistance exercises with an elastic band (Appendix 5, Supplementary Material).

## Discussion

The primary objective of this systematic review was to evaluate the efficacy of conservative interventions for subacromial pain specifically within the older adult population. While our pooled meta-analysis revealed a statistically significant reduction in pain favoring conservative interventions, the effect size was modest and the lower limit of the confidence interval was close to the line of no effect.

For instance, Gialanella et al. [23] demonstrated that augmenting a home-based exercise and monitoring regimen with supervised cycle ergometer training significantly alleviated pain. This aligns with Gutierrez Espinoza et al. [25], who found that preceding joint mobilization with cycle ergometry effectively reduced pain in patients with adhesive capsulitis, suggesting a potential role for systemic aerobic exercise in modulating local shoulder pain.

In the realm of active interventions, Lee et al. [4] investigated the use of elastic band resistance exercises tailored to functional shoulder movements. When compared to a control group maintaining usual care routines, the intervention cohort exhibited marked improvements in pain perception. These results corroborate the earlier findings of Heron et al. [26], who reported comparable reductions in pain and disability following similar resistance-based protocols.

Regarding passive modalities, Calvo-Lobo et al. [6] explored the effects of dry needling applied to latent and active trigger points within the infraspinatus muscle, noting a decrease in both pain intensity and satellite point irritability. However, the absence of a true placebo or an untreated control group severely limits the causal interpretation of these results. This methodological limitation is echoed in the broader literature; as highlighted by prior reviews [27, 28], while dry needling may offer transient, short-term relief—especially when combined with electrical stimulation—it has yet to demonstrate clear superiority over conventional physical therapy.

Similarly exploring passive treatments, Knebl et al. [24] evaluated osteopathic manipulative treatment (OMT) utilizing the Spencer technique against a placebo procedure. The OMT group achieved statistically significant gains in both pain relief and range of motion. These positive outcomes are strongly corroborated by a more recent randomized controlled trial by Iqbal et al. [29], which demonstrated that the Spencer technique yielded statistically superior improvements in pain reduction and functional range of motion when compared to conventional passive stretching.

Despite these individual positive signals, several major methodological limitations hinder a robust synthesis of current evidence. A critical deficiency across the included studies is the pervasive lack of validated, age-appropriate outcome measures to assess upper limb disability, such as the Disabilities of the Arm, Shoulder, and Hand (DASH) questionnaire [30]. Furthermore, although Gialanella et al. [23] employed the Health Assessment Questionnaire to gauge functional capacity, these metrics could not be integrated into our comparative pooled analysis. Compounding this issue is the clinical heterogeneity introduced by some studies focusing on specific underlying pathologies—such as rheumatologic conditions or post-arthroplasty states—which inherently restricts the external validity and generalizability of our findings to the broader older adult population.

Fundamentally, this review exposes a glaring gap in the geriatric rehabilitation literature. Current research predominantly focuses on body structure and function impairments—such as pain intensity and range of motion—while often neglecting functional outcomes, activity limitations, and participation restrictions. Evaluating these higher-order dimensions is vital when addressing musculoskeletal pain in older adults, as functional decline is inextricably linked to increased risks of dependency, morbidity, and diminished overall quality of life.

Consequently, future randomized controlled trials must prioritize patient-centered and functional outcomes. Studies equipped with robust sample sizes, standardized methodological designs, and appropriate control groups are urgently needed to definitively establish the long-term clinical utility of interventions such as resistance training, joint mobilization, and osteopathic techniques in managing subacromial pain in the older people.

## Conclusion

Conservative treatments demonstrate a statistically significant reduction in subacromial pain among older adults. However, given the limited number of studies and the proximity of the confidence interval to the line of no effect, these findings should be interpreted with caution. The current evidence base remains restricted in both quality and quantity, particularly concerning long-term benefits and functional improvements. There is a pressing need for further rigorous research focusing on clinically meaningful outcomes, such as disability, independence, and quality of life, in this specific population.

## Supplementary Information

Below is the link to the electronic supplementary material.

Supplementary file1 (DOCX 33 KB)

Supplementary file2 (DOCX 18 KB)

Supplementary file3 (DOCX 22 KB)

Supplementary file4 (DOCX 15 KB)

Supplementary file5 (DOCX 15 KB)

## Data Availability

All data analyzed during this study are included in this published article [and its supplementary information files - inclua se houver]. No new primary datasets were generated or analyzed.

## Declarations

### Registration

PROSPERO registration number: CRD42024591488.
