# Supplementary material for: Efficacy of treatment for subacromial pain in older adults: a systematic review and meta-analysis with GRADE recommendations
Source: Eur Geriatr Med. 2026 May 28;17(4):2155–62. doi: 10.1007/s41999-026-01511-0 (PMC13562206; doi:10.1007/s41999-026-01511-0)
Supplement: Supplementary file 2 [file 41999_2026_1511_MOESM2_ESM.docx]

Appendix 2. Search Strategy Conducted up to July 12th, 2023 (updated July 22, 2025).

OVID (MEDLINE, COCHRANE, EMBASE, AMED, PSYCINFO)

1. randomised controlled trial*.mp. [mp=ab, hw, ti, tn, ot, dm, mf, dv, kf, fx, dq, bt, nm, ox, px, rx, ui, sy, ux, mx, tc, id, tm, sh, kw, tx, ct]
2. Randomized Controlled Trial.mp. [mp=ab, hw, ti, tn, ot, dm, mf, dv, kf, fx, dq, bt, nm, ox, px, rx, ui, sy, ux, mx, tc, id, tm, sh, kw, tx, ct]
3. random allocation.mp. [mp=ab, hw, ti, tn, ot, dm, mf, dv, kf, fx, dq, bt, nm, ox, px, rx, ui, sy, ux, mx, tc, id, tm, sh, kw, tx, ct]
4. Comparative Stud*.mp. [mp=ab, hw, ti, tn, ot, dm, mf, dv, kf, fx, dq, bt, nm, ox, px, rx, ui, sy, ux, mx, tc, id, tm, sh, kw, tx, ct]
5. Controlled Clinical Trial*.mp. [mp=ab, hw, ti, tn, ot, dm, mf, dv, kf, fx, dq, bt, nm, ox, px, rx, ui, sy, ux, mx, tc, id, tm, sh, kw, tx, ct]
6. double-blind method*.mp. [mp=ab, hw, ti, tn, ot, dm, mf, dv, kf, fx, dq, bt, nm, ox, px, rx, ui, sy, ux, mx, tc, id, tm, sh, kw, tx, ct]
7. single-blind method*.mp. [mp=ab, hw, ti, tn, ot, dm, mf, dv, kf, fx, dq, bt, nm, ox, px, rx, ui, sy, ux, mx, tc, id, tm, sh, kw, tx, ct]
8. Clinical Trial*.mp. [mp=ab, hw, ti, tn, ot, dm, mf, dv, kf, fx, dq, bt, nm, ox, px, rx, ui, sy, ux, mx, tc, id, tm, sh, kw, tx, ct]
9. crossover stud*.mp. [mp=ab, hw, ti, tn, ot, dm, mf, dv, kf, fx, dq, bt, nm, ox, px, rx, ui, sy, ux, mx, tc, id, tm, sh, kw, tx, ct]
10. 1 or 2 or 3 or 4 or 5 or 6 or 7 or 8 or 9
11. shoulder pain.mp. [mp=ab, hw, ti, tn, ot, dm, mf, dv, kf, fx, dq, bt, nm, ox, px, rx, ui, sy, ux, mx, tc, id, tm, sh, kw, tx, ct]
12. shoulder tendin*.mp. [mp=ab, hw, ti, tn, ot, dm, mf, dv, kf, fx, dq, bt, nm, ox, px, rx, ui, sy, ux, mx, tc, id, tm, sh, kw, tx, ct]
13. supraspinatus tendin*.mp. [mp=ab, hw, ti, tn, ot, dm, mf, dv, kf, fx, dq, bt, nm, ox, px, rx, ui, sy, ux, mx, tc, id, tm, sh, kw, tx, ct]
14. infraspinatus tendin*.mp. [mp=ab, hw, ti, tn, ot, dm, mf, dv, kf, fx, dq, bt, nm, ox, px, rx, ui, sy, ux, mx, tc, id, tm, sh, kw, tx, ct]
15. teres minor tendin*.mp. [mp=ab, hw, ti, tn, ot, dm, mf, dv, kf, fx, dq, bt, nm, ox, px, rx, ui, sy, ux, mx, tc, id, tm, sh, kw, tx, ct]
16. subscapularis tendin*.mp. [mp=ab, hw, ti, tn, ot, dm, mf, dv, kf, fx, dq, bt, nm, ox, px, rx, ui, sy, ux, mx, tc, id, tm, sh, kw, tx, ct]
17. bicip* tendin*.mp. [mp=ab, hw, ti, tn, ot, dm, mf, dv, kf, fx, dq, bt, nm, ox, px, rx, ui, sy, ux, mx, tc, id, tm, sh, kw, tx, ct]
18. rotator cuff tendin*.mp. [mp=ab, hw, ti, tn, ot, dm, mf, dv, kf, fx, dq, bt, nm, ox, px, rx, ui, sy, ux, mx, tc, id, tm, sh, kw, tx, ct]
19. tendin*.mp. [mp=ab, hw, ti, tn, ot, dm, mf, dv, kf, fx, dq, bt, nm, ox, px, rx, ui, sy, ux, mx, tc, id, tm, sh, kw, tx, ct]
20. subacromial pain syndrome.mp. [mp=ab, hw, kw, ti, ot, tn, dm, mf, dv, kf, fx, dq, bt, nm, ox, px, rx, an, ui, sy, ux, mx, tc, id, tm, sh, tx, ct]
21. subacromial pain.mp. [mp=ab, hw, kw, ti, ot, tn, dm, mf, dv, kf, fx, dq, bt, nm, ox, px, rx, an, ui, sy, ux, mx, tc, id, tm, sh, tx, ct]
22. Shoulder Impingement Syndrome.mp. [mp=ab, hw, kw, ti, ot, tn, dm, mf, dv, kf, fx, dq, bt, nm, ox, px, rx, an, ui, sy, ux, mx, tc, id, tm, sh, tx, ct]
23. shoulder bursitis.mp. [mp=ab, hw, kw, ti, ot, tn, dm, mf, dv, kf, fx, dq, bt, nm, ox, px, rx, an, ui, sy, ux, mx, tc, id, tm, sh, tx, ct]
24. 11 or 12 or 13 or 14 or 15 or 16 or 17 or 18 or 19 or 20 or 21 or 22 or 23
25. aged.mp. [mp=ab, hw, kw, ti, ot, tn, dm, mf, dv, kf, fx, dq, bt, nm, ox, px, rx, an, ui, sy, ux, mx, tc, id, tm, sh, tx, ct]
26. aging.mp. [mp=ab, hw, kw, ti, ot, tn, dm, mf, dv, kf, fx, dq, bt, nm, ox, px, rx, an, ui, sy, ux, mx, tc, id, tm, sh, tx, ct]
27. older*.mp. [mp=ab, hw, kw, ti, ot, tn, dm, mf, dv, kf, fx, dq, bt, nm, ox, px, rx, an, ui, sy, ux, mx, tc, id, tm, sh, tx, ct]
28. elder*.mp. [mp=ab, hw, kw, ti, ot, tn, dm, mf, dv, kf, fx, dq, bt, nm, ox, px, rx, an, ui, sy, ux, mx, tc, id, tm, sh, tx, ct]
29. ancient*.mp. [mp=ab, hw, kw, ti, ot, tn, dm, mf, dv, kf, fx, dq, bt, nm, ox, px, rx, an, ui, sy, ux, mx, tc, id, tm, sh, tx, ct]
30. grey haired.mp. [mp=ab, hw, kw, ti, ot, tn, dm, mf, dv, kf, fx, dq, bt, nm, ox, px, rx, an, ui, sy, ux, mx, tc, id, tm, sh, tx, ct]
31. advanced in years.mp. [mp=ab, hw, kw, ti, ot, tn, dm, mf, dv, kf, fx, dq, bt, nm, ox, px, rx, an, ui, sy, ux, mx, tc, id, tm, sh, tx, ct]
32. 25 or 26 or 27 or 28 or 29 or 30 or 31
33. 10 and 24 and 32

PEDro

Abstract & Title: shoulder pain Therapy: not applicable Problem: pain

Body Part: upper arm, shoulder, or shoulder girdle Subdiscipline: musculoskeletal

Topic: not applicable Method: clinical trial

Author/Association: not applicable Title Only: not applicable

Source: not applicable Published Since: not applicable

New records added since: not applicable Score of at least: not applicable
