# Supplementary material for: Efficacy of treatment for subacromial pain in older adults: a systematic review and meta-analysis with GRADE recommendations
Source: Eur Geriatr Med. 2026 May 28;17(4):2155–62. doi: 10.1007/s41999-026-01511-0 (PMC13562206; doi:10.1007/s41999-026-01511-0)
Supplement: Supplementary file 3 [file 41999_2026_1511_MOESM3_ESM.docx]

Table 1- PICOT research question

**Patient**	Older adults with subacromial pain

**Intervention**	Exercise for treatment

**Comparator**	Minimal or no interventions (e.g. placebo, sham, or wait list).

**Outcomes**	Pain intensity or disability

**Time**	Short, medium, and long-term effects
