# Supplementary material for: Efficacy of treatment for subacromial pain in older adults: a systematic review and meta-analysis with GRADE recommendations
Source: Eur Geriatr Med. 2026 May 28;17(4):2155–62. doi: 10.1007/s41999-026-01511-0 (PMC13562206; doi:10.1007/s41999-026-01511-0)
Supplement: Supplementary file 4 [file 41999_2026_1511_MOESM4_ESM.docx]

Table 3: Characterization of pain

| **Study** | **Scale** | **Central tendency IG** | **Variability IG** | **Sample Size IG** | **Central tendency CG** | **Variability CG** | **Sample Size CG** |
| --- | --- | --- | --- | --- | --- | --- | --- |
| Calvo-Lobo et al ., 2018 | NRS (0 a 10) | 3.03 | 1.51 | 32 | 3.13 | 1.61 | 31 |
| Gialanella et al ., 2018 | VNS (0 a 10) | 4.57 | 1.95 | 19 | 6.94 | 1.5 | 19 |
| Lee et al ., 2022 | NRS (0 a 10) | 1.66 | 0.93 | 38 | 4.03 | 1.72 | 39 |
