# Supplementary material for: Efficacy of treatment for subacromial pain in older adults: a systematic review and meta-analysis with GRADE recommendations
Source: Eur Geriatr Med. 2026 May 28;17(4):2155–62. doi: 10.1007/s41999-026-01511-0 (PMC13562206; doi:10.1007/s41999-026-01511-0)
Supplement: Supplementary file 5 [file 41999_2026_1511_MOESM5_ESM.docx]

**Question::** [Exercise for treatment] compared to [control] to [Elderly with supracromial pain]

| **Certainty assessment** | | | | | | | **№ of patients** | | **Effect** | | **Certainty** | **Importance** |
| --- | --- | --- | --- | --- | --- | --- | --- | --- | --- | --- | --- | --- |
| **№ of studies** | **Study design** | **Risk of bias** | **Inconsistency** | **Indirectness** | **Imprecision** | **Other considerations** | **[respiratory muscle training]** | **[control group]** | **Relative (95% CI)** | **Absolute (95% CI)** |  |  |
| **pain (avaliado com: NRS (0 a 10))** | | | | | | | | | | | | |
| 3 | randomised trials | serious^a,b^ | serious^c^ | not serious | not serious | none | 89 | 89 | - | MD 1.6 higher (3.16 higher to 0.04 higher) | ⨁⨁◯◯ low |  |

**CI:** Confidence interval

#### Explanations

a. b. b. Estimative of effect varied across studies and I² for direct comparisons was moderate (30-60%)

b. c. c. The wide confidence intervals cross the threshold for an important benefit, no benefit, and harm

c. a. a. The direct estimate showed more than 50% of trials favoring the treatment were judged to have some concerns in randomization and blinding and these criteria are important for outcome evaluated b.
